# Crystal structure of 4-amino­benzoic acid–4-methyl­pyridine (1/1)

**DOI:** 10.1107/S2056989015000791

**Published:** 2015-01-21

**Authors:** M. Krishna Kumar, P. Pandi, S. Sudhahar, G. Chakkaravarthi, R. Mohan Kumar

**Affiliations:** a Department of Physics, Presidency College, Chennai 600 005, India; b Department of Physics, Panimalar Engineering College, Chennai 600 123, India; c Department of Physics, CPCL Polytechnic College, Chennai 600 068, India

**Keywords:** crystal structure, adduct, O—H⋯N and N—H⋯O hydrogen bonds, layered structure

## Abstract

In the title 1:1 adduct, C_6_H_7_N·C_7_H_7_NO_2_, the carb­oxy­lic acid group is twisted at an angle of 4.32 (18)° with respect to the attached benzene ring. In the crystal, the carb­oxy­lic acid group is linked to the pyridine ring by an O—H⋯N hydrogen bond, forming a dimer. The dimers are linked by N—H⋯O hydrogen bonds, generating (010) sheets.

## Related literature   

For background to pyridine derivatives, see: Tomaru *et al.* (1991 ▸). Katritzky *et al.* (1996 ▸); Akkurt *et al.* (2005 ▸). For related structures, see: Smith & Wermuth (2010 ▸); Hemamalini & Fun (2010 ▸); Kannan *et al.* (2012 ▸); Thanigaimani *et al.* (2012 ▸); Muralidharan *et al.* (2013 ▸).
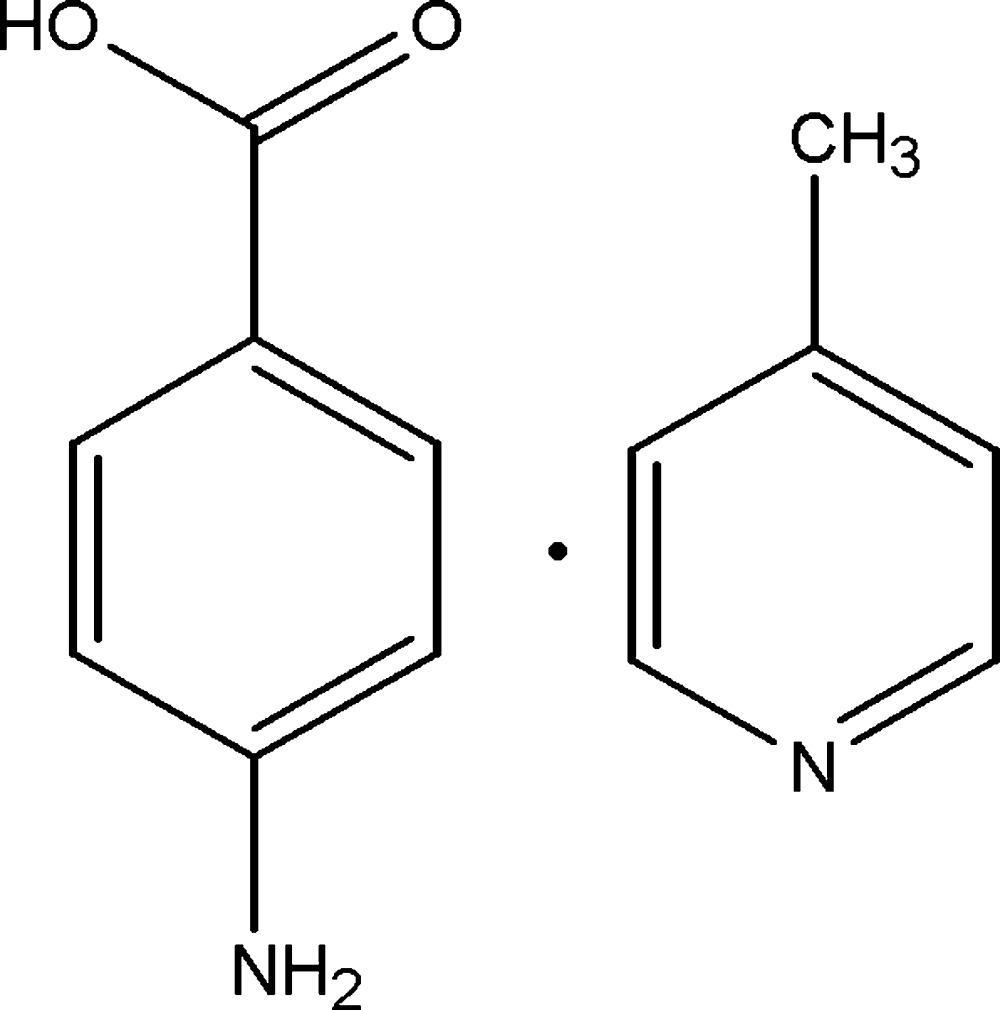


## Experimental   

### Crystal data  

C_6_H_7_N·C_7_H_7_NO_2_
*M* *_r_* = 230.26
Monoclinic, 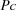

*a* = 7.5970 (7) Å
*b* = 11.6665 (12) Å
*c* = 7.6754 (8) Å
β = 114.200 (3)°
*V* = 620.49 (11) Å^3^
*Z* = 2
Mo *K*α radiation
μ = 0.09 mm^−1^
*T* = 295 K
0.28 × 0.24 × 0.20 mm

### Data collection  

Bruker Kappa APEXII CCD diffractometer
Absorption correction: multi-scan (*SADABS*; Sheldrick, 1996 ▸) *T*_min_ = 0.977, *T*_max_ = 0.983
10064 measured reflections
2144 independent reflections
1458 reflections with *I* > 2σ(*I*)
*R*_int_ = 0.030

### Refinement  

*R*[*F* ^2^ > 2σ(*F* ^2^)] = 0.038
*wR*(*F* ^2^) = 0.108
*S* = 1.03
2144 reflections
159 parameters
3 restraints
H atoms treated by a mixture of independent and constrained refinement
Δρ_max_ = 0.12 e Å^−3^
Δρ_min_ = −0.13 e Å^−3^

### 

Data collection: *APEX2* (Bruker, 2004 ▸); cell refinement: *SAINT* (Bruker, 2004 ▸); data reduction: *SAINT*; program(s) used to solve structure: *SHELXS97* (Sheldrick, 2008 ▸); program(s) used to refine structure: *SHELXL97* (Sheldrick, 2015 ▸); molecular graphics: *PLATON* (Spek, 2009 ▸); software used to prepare material for publication: *SHELXL97*.

## Supplementary Material

Crystal structure: contains datablock(s) global, I. DOI: 10.1107/S2056989015000791/hb7348sup1.cif

Structure factors: contains datablock(s) I. DOI: 10.1107/S2056989015000791/hb7348Isup2.hkl

**Fig. 1.**
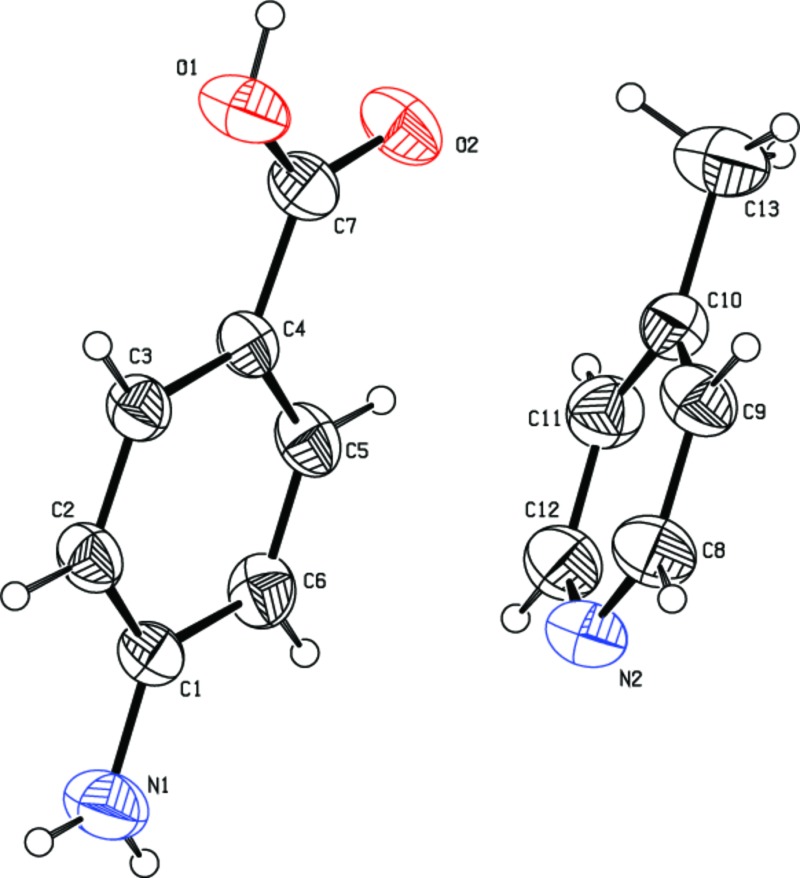
The molecular structure of (I), with 30% probability displacement ellipsoids for non-H atoms.

. DOI: 10.1107/S2056989015000791/hb7348fig1.tif
The mol­ecular structure of (I), with 30% probability displacement ellipsoids for non-H atoms.

CCDC reference: 1043592

Additional supporting information:  crystallographic information; 3D view; checkCIF report

## Figures and Tables

**Table 1:** Hydrogen-bond geometry (, )

*D*H*A*	*D*H	H*A*	*D* *A*	*D*H*A*
O1H1N2^i^	0.84(1)	1.81(1)	2.644(3)	177(4)
N1H1*A*O2^ii^	0.86	2.32	3.049(3)	142
N1H1*B*O2^iii^	0.86	2.17	3.031(3)	174

Symmetry codes: (i) 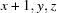; (ii) 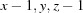; (iii) 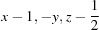.

## supplementary crystallographic information

### S1. Chemical context

Amino­pyridine and its derivatives play an important role in heterocyclic chemistry (Katritzky *et al.*, 1996). Some pyridine derivatives possess nonlinear optical (NLO) properties (Tomaru *et al.*, 1991) and possess anti­bacterial and anti­fungal activities (Akkurt *et al.*, 2005). we herewith, report the synthesis and the crystal structure of (I) (Fig. 1).

### S2. Structural commentary

The molecular structure of the title compound (I) is shown in (Fig. 1). It consists of two independent molecules in the assymetric unit. In the 4-amino­benzoic acid molecule, the carboxyl group is twisted at an angle of 4.32 (18)° with respect to the aromatic ring. In the 4-methyl­pyridine molecule, the pyridine ring (C8—C12/N2) is almost planar [maximum deviation 0.002 (3) Å]. The dihedral angle between the benzene ring (C1—C6) and pyridine ring (C8—C12/N2) is 57.11 (14)°.

### S3. Supra­molecular features

In the crystal structure, 4-amino­benzoate and 4-methyl­pyridine molecules are linked by weak inter­molecular O—H···N hydrogen bonds and forms infinite one-dimensional chain along [0 0 1]. The adjacent 4-amino­benzoate molecules are connected by weak inter­molecular N—H···O hydrogen bonds, forming R^2^_2_(12) ring motif in a two-dimensional network in the (010) plane (Table 2 & Fig. 2).

### S4. Database survey

Several similar structures containing methyl­pyridinium and nitro­benzoate molecules have been reported earlier: i.e., 2-Amino-5-methyl­pyridinium 2-amino­benzoate (Thanigaimani *et al.*, 2012); 2-Amino-5-chloro­pyridinium 4-amino­benzoate (Kannan *et al.*, 2012); 2-Amino-4-methyl­pyridinium 2-nitro­benzoate (Muralidharan *et al.*, 2013); 4-Methyl­pyridinium 2-carb­oxy-4,5-di­chloro­benzoate monohydrate [Smith & Wermuth, (2010)]; 2-Amino-4-methyl­pyridinium 2-hy­droxy­benzoate [Hemamalini & Fun (2010)].

### S5. Synthesis and crystallization

Equimolar qu­antity of 4-methyl­pyridine and 4-amino­benzoic acid were dissolved in methanol-water mixed solvent and colourless blocks of the title adduct were grown by slow evaporation of the solvents.

### S6. Refinement

Crystal data, data collection and structure refinement details are summarized in Table 1. The hydrogen atoms attached to C atoms and N atom were fixed geometrically and treated as riding with C—H = 0.93 Å (aromatic) or 0.96 Å (methyl) and N—H = 0.86 Å with Uiso(H) = 1.2 Ueq(C or N) or 1.5 Ueq(C) The hydroxyl H atom was located in a difference Fourier map, and refined with Uiso(H) = 1.2 Ueq(O) and distance restraint O—H = 0.82 Å.

### Crystal data

**Table:**

C_6_H_7_N·C_7_H_7_NO_2_	*F*(000) = 244
*M**_r_* = 230.26	*D*_x_ = 1.232 Mg m^−^^3^
Monoclinic, *P**c*	Mo *K*α radiation, λ = 0.71073 Å
Hall symbol: P -2yc	Cell parameters from 2749 reflections
*a* = 7.5970 (7) Å	θ = 3.4–21.8°
*b* = 11.6665 (12) Å	µ = 0.09 mm^−^^1^
*c* = 7.6754 (8) Å	*T* = 295 K
β = 114.200 (3)°	Block, colourless
*V* = 620.49 (11) Å^3^	0.28 × 0.24 × 0.20 mm
*Z* = 2	

### Data collection

**Table:**

Bruker Kappa APEXII CCD diffractometer	2144 independent reflections
Radiation source: fine-focus sealed tube	1458 reflections with *I* > 2σ(*I*)
Graphite monochromator	*R*_int_ = 0.030
ω and φ scan	θ_max_ = 26.7°, θ_min_ = 3.4°
Absorption correction: multi-scan (*SADABS*; Sheldrick, 1996)	*h* = −9→8
*T*_min_ = 0.977, *T*_max_ = 0.983	*k* = −14→14
10064 measured reflections	*l* = −9→9

### Refinement

**Table:**

Refinement on *F*^2^	Secondary atom site location: difference Fourier map
Least-squares matrix: full	Hydrogen site location: inferred from neighbouring sites
*R*[*F*^2^ > 2σ(*F*^2^)] = 0.038	H atoms treated by a mixture of independent and constrained refinement
*wR*(*F*^2^) = 0.108	*w* = 1/[σ^2^(*F*_o_^2^) + (0.0554*P*)^2^ + 0.0229*P*] where *P* = (*F*_o_^2^ + 2*F*_c_^2^)/3
*S* = 1.03	(Δ/σ)_max_ < 0.001
2144 reflections	Δρ_max_ = 0.12 e Å^−^^3^
159 parameters	Δρ_min_ = −0.13 e Å^−^^3^
3 restraints	Extinction correction: *SHELXL97* (Sheldrick, 2008), Fc^*^=kFc[1+0.001xFc^2^λ^3^/sin(2θ)]^-1/4^
Primary atom site location: structure-invariant direct methods	Extinction coefficient: 0.013 (4)

### Special details

**Table:**

Geometry. All esds (except the esd in the dihedral angle between two l.s. planes) are estimated using the full covariance matrix. The cell esds are taken into account individually in the estimation of esds in distances, angles and torsion angles; correlations between esds in cell parameters are only used when they are defined by crystal symmetry. An approximate (isotropic) treatment of cell esds is used for estimating esds involving l.s. planes.
Refinement. Refinement of F^2^ against ALL reflections. The weighted R-factor wR and goodness of fit S are based on F^2^, conventional R-factors R are based on F, with F set to zero for negative F^2^. The threshold expression of F^2^ > 2sigma(F^2^) is used only for calculating R-factors(gt) etc. and is not relevant to the choice of reflections for refinement. R-factors based on F^2^ are statistically about twice as large as those based on F, and R- factors based on ALL data will be even larger.

### Fractional atomic coordinates and isotropic or equivalent isotropic displacement parameters (Å^2^)

**Table:**

	*x*	*y*	*z*	*U*_iso_*/*U*_eq_	
C1	0.3693 (4)	0.1034 (2)	0.7149 (4)	0.0567 (6)	
C2	0.4434 (4)	0.2029 (2)	0.6722 (4)	0.0591 (7)	
H2	0.3671	0.2471	0.5675	0.071*	
C3	0.6273 (4)	0.2362 (2)	0.7829 (3)	0.0558 (6)	
H3	0.6737	0.3037	0.7529	0.067*	
C4	0.7475 (4)	0.1730 (2)	0.9383 (4)	0.0508 (6)	
C5	0.6737 (4)	0.0738 (2)	0.9798 (4)	0.0594 (7)	
H5	0.7516	0.0294	1.0835	0.071*	
C6	0.4892 (4)	0.0392 (2)	0.8724 (4)	0.0629 (7)	
H6	0.4429	−0.0278	0.9043	0.076*	
C7	0.9416 (4)	0.2104 (2)	1.0596 (4)	0.0587 (7)	
C8	0.4371 (5)	0.4546 (2)	1.3188 (5)	0.0729 (8)	
H8	0.3567	0.5148	1.3183	0.087*	
C9	0.6306 (4)	0.4655 (2)	1.4263 (4)	0.0693 (8)	
H9	0.6791	0.5319	1.4967	0.083*	
C10	0.7534 (4)	0.3784 (2)	1.4304 (4)	0.0639 (7)	
C11	0.6716 (4)	0.2836 (2)	1.3242 (4)	0.0718 (8)	
H11	0.7487	0.2220	1.3229	0.086*	
C12	0.4767 (5)	0.2790 (3)	1.2196 (4)	0.0766 (9)	
H12	0.4249	0.2135	1.1479	0.092*	
C13	0.9658 (5)	0.3866 (3)	1.5468 (6)	0.0954 (11)	
H13A	1.0310	0.3941	1.4635	0.143*	
H13B	1.0099	0.3186	1.6229	0.143*	
H13C	0.9930	0.4524	1.6289	0.143*	
N1	0.1840 (4)	0.0710 (2)	0.6087 (4)	0.0827 (8)	
H1A	0.1113	0.1124	0.5137	0.099*	
H1B	0.1395	0.0092	0.6364	0.099*	
N2	0.3582 (3)	0.3627 (2)	1.2152 (4)	0.0722 (6)	
O1	0.9921 (3)	0.30989 (17)	1.0123 (3)	0.0808 (6)	
H1	1.109 (2)	0.324 (3)	1.076 (5)	0.121*	
O2	1.0516 (3)	0.15755 (17)	1.1996 (3)	0.0778 (6)	

### Atomic displacement parameters (Å^2^)

**Table:**

	*U*^11^	*U*^22^	*U*^33^	*U*^12^	*U*^13^	*U*^23^
C1	0.0545 (16)	0.0584 (15)	0.0526 (15)	−0.0071 (14)	0.0173 (13)	−0.0070 (14)
C2	0.0599 (17)	0.0581 (15)	0.0489 (15)	0.0009 (12)	0.0118 (13)	0.0088 (12)
C3	0.0582 (16)	0.0536 (13)	0.0509 (15)	−0.0050 (13)	0.0175 (13)	0.0058 (12)
C4	0.0529 (14)	0.0490 (12)	0.0442 (13)	0.0040 (12)	0.0135 (12)	0.0026 (12)
C5	0.0671 (18)	0.0516 (14)	0.0484 (16)	0.0021 (13)	0.0124 (14)	0.0046 (12)
C6	0.077 (2)	0.0518 (14)	0.0590 (17)	−0.0076 (14)	0.0273 (15)	0.0048 (13)
C7	0.0563 (17)	0.0532 (13)	0.0583 (17)	0.0045 (13)	0.0150 (14)	0.0003 (14)
C8	0.0630 (18)	0.0626 (16)	0.081 (2)	0.0031 (15)	0.0172 (16)	0.0007 (16)
C9	0.068 (2)	0.0617 (17)	0.0665 (19)	−0.0085 (14)	0.0150 (16)	−0.0042 (13)
C10	0.0579 (18)	0.0740 (17)	0.0585 (17)	−0.0045 (16)	0.0225 (14)	0.0063 (15)
C11	0.0680 (19)	0.0723 (18)	0.078 (2)	0.0015 (15)	0.0325 (18)	−0.0048 (16)
C12	0.077 (2)	0.0747 (19)	0.074 (2)	−0.0158 (17)	0.0260 (17)	−0.0171 (16)
C13	0.0604 (19)	0.105 (2)	0.104 (3)	−0.0070 (17)	0.0163 (18)	0.002 (2)
N1	0.0655 (16)	0.0852 (18)	0.0809 (18)	−0.0163 (13)	0.0132 (14)	0.0063 (15)
N2	0.0567 (14)	0.0719 (15)	0.0766 (16)	−0.0059 (13)	0.0157 (12)	−0.0023 (13)
O1	0.0588 (11)	0.0691 (12)	0.0883 (16)	−0.0105 (10)	0.0035 (11)	0.0130 (11)
O2	0.0678 (13)	0.0726 (12)	0.0665 (12)	0.0068 (10)	0.0006 (10)	0.0090 (10)

### Geometric parameters (Å, º)

**Table:**

C1—N1	1.359 (4)	C8—H8	0.9300
C1—C2	1.387 (3)	C9—C10	1.371 (4)
C1—C6	1.397 (4)	C9—H9	0.9300
C2—C3	1.361 (4)	C10—C11	1.363 (4)
C2—H2	0.9300	C10—C13	1.493 (5)
C3—C4	1.380 (3)	C11—C12	1.366 (4)
C3—H3	0.9300	C11—H11	0.9300
C4—C5	1.379 (3)	C12—N2	1.319 (4)
C4—C7	1.452 (3)	C12—H12	0.9300
C5—C6	1.364 (4)	C13—H13A	0.9600
C5—H5	0.9300	C13—H13B	0.9600
C6—H6	0.9300	C13—H13C	0.9600
C7—O2	1.224 (3)	N1—H1A	0.8600
C7—O1	1.319 (3)	N1—H1B	0.8600
C8—N2	1.323 (3)	O1—H1	0.836 (10)
C8—C9	1.365 (4)		
			
N1—C1—C2	120.8 (2)	C8—C9—C10	119.9 (3)
N1—C1—C6	121.2 (2)	C8—C9—H9	120.0
C2—C1—C6	118.0 (2)	C10—C9—H9	120.0
C3—C2—C1	120.2 (2)	C11—C10—C9	116.6 (3)
C3—C2—H2	119.9	C11—C10—C13	121.7 (3)
C1—C2—H2	119.9	C9—C10—C13	121.7 (3)
C2—C3—C4	122.2 (2)	C10—C11—C12	120.2 (3)
C2—C3—H3	118.9	C10—C11—H11	119.9
C4—C3—H3	118.9	C12—C11—H11	119.9
C5—C4—C3	117.4 (2)	N2—C12—C11	123.4 (3)
C5—C4—C7	120.4 (2)	N2—C12—H12	118.3
C3—C4—C7	122.1 (2)	C11—C12—H12	118.3
C6—C5—C4	121.5 (2)	C10—C13—H13A	109.5
C6—C5—H5	119.2	C10—C13—H13B	109.5
C4—C5—H5	119.2	H13A—C13—H13B	109.5
C5—C6—C1	120.6 (2)	C10—C13—H13C	109.5
C5—C6—H6	119.7	H13A—C13—H13C	109.5
C1—C6—H6	119.7	H13B—C13—H13C	109.5
O2—C7—O1	120.9 (3)	C1—N1—H1A	120.0
O2—C7—C4	124.1 (2)	C1—N1—H1B	120.0
O1—C7—C4	115.0 (2)	H1A—N1—H1B	120.0
N2—C8—C9	123.3 (3)	C12—N2—C8	116.6 (3)
N2—C8—H8	118.4	C7—O1—H1	112 (3)
C9—C8—H8	118.4		
			
N1—C1—C2—C3	178.1 (3)	C3—C4—C7—O2	179.2 (3)
C6—C1—C2—C3	−0.5 (4)	C5—C4—C7—O1	−176.1 (2)
C1—C2—C3—C4	0.9 (4)	C3—C4—C7—O1	1.3 (3)
C2—C3—C4—C5	−0.5 (4)	N2—C8—C9—C10	0.0 (5)
C2—C3—C4—C7	−178.1 (2)	C8—C9—C10—C11	−0.2 (4)
C3—C4—C5—C6	−0.2 (4)	C8—C9—C10—C13	−179.7 (3)
C7—C4—C5—C6	177.4 (2)	C9—C10—C11—C12	0.4 (4)
C4—C5—C6—C1	0.5 (4)	C13—C10—C11—C12	179.9 (3)
N1—C1—C6—C5	−178.8 (3)	C10—C11—C12—N2	−0.4 (5)
C2—C1—C6—C5	−0.2 (4)	C11—C12—N2—C8	0.1 (5)
C5—C4—C7—O2	1.7 (4)	C9—C8—N2—C12	0.1 (5)

### Hydrogen-bond geometry (Å, º)

**Table:**

*D*—H···*A*	*D*—H	H···*A*	*D*···*A*	*D*—H···*A*
O1—H1···N2^i^	0.84 (1)	1.81 (1)	2.644 (3)	177 (4)
N1—H1*A*···O2^ii^	0.86	2.32	3.049 (3)	142
N1—H1*B*···O2^iii^	0.86	2.17	3.031 (3)	174

Symmetry codes: (i) *x*+1, *y*, *z*; (ii) *x*−1, *y*, *z*−1; (iii) *x*−1, −*y*, *z*−1/2.
