# Supplementary figures and images for: Crystal structure of 4-amino­benzoic acid–4-methyl­pyridine (1/1)
Source: Acta Crystallogr E Crystallogr Commun. 2015 Jan 21;71(Pt 2):o125–6. doi: 10.1107/S2056989015000791 (PMC4384565; doi:10.1107/S2056989015000791)

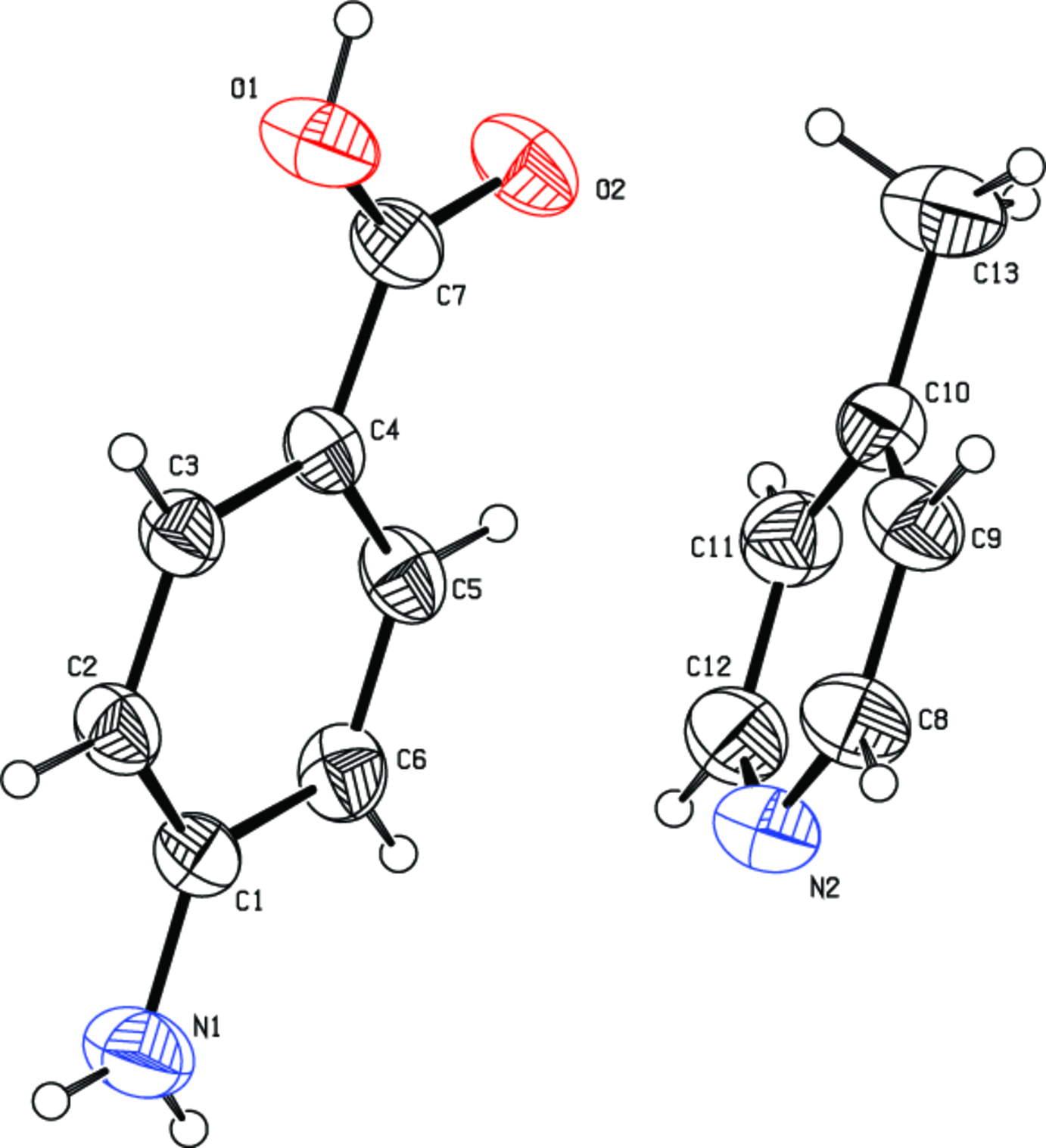

Supplement: Supplementary file 3 [file e-71-0o125-fig1.tif]
